# Production of Non-specific Germicidal Substances by Means of Anti-staphylococcic and Anti-streptococcic Vaccines

**Published:** 1919

**Authors:** 


**Production of Non-specific Germicidal Substances by Means of Anti-staphylococcic and Anti-streptococcic Vaccines.** By Sir ALMROTH E. WRIGHT. *Comptes Rendus de l'Académie des Sciences*, October 21, 1918.

The micro-organisms existing in infected wounds are classified by the author as " serosaprophytes " and " serophytes ".

Among the *serosaprophyte* varieties are most of the common micro-organisms. The blood serum and lymph exert a marked germicidal action upon these bacilli; their culture in such media is impossible.

On the contrary, the normal serum is an excellent medium for culturing the *serophyte* varieties, among which are the staphylococcus and streptococcus.

Consequently, the destruction of staphylococci and streptococci in the normal blood can only be obtained by means of the phagocytes. This destruction can be repeated in vitro, either by true phagocytosis, or, if great care is taken to remove the last drops of serum, by bactericidal substances, exuding from the living leukocytes, in contact with the bacilli.

The author has demonstrated that non-specific germicidal substances develop in the blood subsequent to the action of antistaphylococcic and antistreptococcic vaccines.

The tests have been carried out on rabbits; the vaccines were injected into the jugular veins; small quantities of the circulating blood were examined every half-hour after the injection.

A non-specific bactericidal power develops in the blood serum very soon after the injection of proper doses of vaccines, and this action increases for several hours after the injection.

The same tests were undertaken " in vitro ". The vaccine was mixed directly with a certain amount of blood removed from the organism, and diluted with normal saline. The whole mixture was heated moderately and constantly for two hours and a half; its germicidal power was then tested by trying to grow in it both staphylococci and streptococci. The cultures obtained were examined microscopically after 24 hours' growth.

*Results.* -- Non-specific bactericidal substances develop in these experiments, their development depending above all on the dose of vaccine used. The same results are obtained both " in vivo " and " in vitro ".

All serous fluids of the organism acquire this germicidal power and it lasts there as long as 24 and 48 hours.

The author suggests that henceforth injected patients should be transfused with immune blood, in which this germicidal power should have developed by previous contact with proper doses of vaccine.

The quantity of vaccine which, acting upon a certain amount of blood, develops therein the highest germicidal power, should be determined experimentally in each case, before resorting to any injection of vaccine.

